# The association between aortic stiffness parameters and left ventricular deformation: preliminary results from the MESA 5 study

**DOI:** 10.1186/1532-429X-14-S1-O46

**Published:** 2012-02-01

**Authors:** Atul R Chugh, Kihei Yoneyama, Ola Gjesdal, Vishal C Mehra, Gisela Teixido-Tura, Doris Chen, Chia-Ying Liu, Alban Redheuil, David A Bluemke, Joao A Lima

**Affiliations:** 1 Cardiovascular Medicine, Johns Hopkins University, Baltimore, MD, USA; 2 Cardiovascular Imaging, National Institute of Health, Bethesda, MD, USA

## Background

Aortic stiffness parameters have been studied as a risk marker corollary to traditional risk markers used in Framingham risk stratification. Differences in risk prediction ability have been noted between pulse wave velocity (PWV) and other parameters. Here, the relationship of PWV and aortic distensibility with left ventricular deformational measurements (e.g. radial strain, circumferential strain, ejection fraction and torsion) are explored.

## Methods

Cardiac MR studies from the MESA 5 cohort were consecutively selected for aortic stiffness and LV deformational measurements. Studies were performed using 1.5-T whole-body MRI systems and phase contrast cine gradient echo sequence with ECG gating to evaluate aortic size and stiffness. Transverse views were used for luminal and flow measurements while parasagittal (candy cane) views were used for aortic arch length measurements. Aorta analyses were performed using a validated, automated software package (ARTFUN; INSERM U678). LV mass and ejection fraction and radial strain measurements were evaluated based on cine short axis views and 2-chamber, 4-chamber and 3-chamber views using the Cardiac Image Modeller (CIM, Auckland, New Zealand) and circumferential strain and torsion were assessed based on tagged short axis images using Harmonic Phase Imaging (HARP, Diagnosoft, North Carolina). Univariate analyses of parameters were performed using Pearson’s Product Moment Correlational Analysis while binary variable analyses were performed using ANOVA. Variables approaching significance were then examined for multifactorial correlation using linear regression models.

## Results

Aortic and LV deformation analyses were performed in 221 cases. Mean age was 69.4±8.4 years, 58.2% were female and 44.4% were non-Caucasian. Mean LV ejection fraction was 61.7 ± 8.3% while mean LV mass was 134.6 ± 30.5 g. Univariate analyses of aortic parameters revealed significant association between PWV and age (r=0.252, p<0.001), mid-wall circumferential strain (r=0.173, p=0.01) and average radial strain (r=-0.181,p=0.043) while aortic distensibility was associated with ejection fraction (r=0.184, p=0.039) and radial strain (r=0.218,p=0.014) alone. In multivariate linear regression models, only age remained significant (β=.268, p=0.004) for PWV while torsion showed a trend for significance (β=.178, p=0.091). For distensibility, only mid-wall circumferential strain (β=-0.275, p=0.004) was significant while a trend was noted for average radial strain (β=0.185, p=0.147).

## Conclusions

Associations between aortic distensibility and PWV with LV deformational parameters are different in that PWV is more strongly associated with age while distensibility is linked with LV deformational parameters. Mechanisms governing changes in aortic stiffness may differ for each parameter. Biomechanical influences affecting each aortic parameter also differ. Further characterization of these changes in longitudinal fashion is warranted using clinical data.

## Funding

National Institutes of Health-National Heart, Lung, and Blood Institute.

